# Endometriosis Unveiled: A Tale of Two Extremes, From Acute Sepsis to Silent Suspicion

**DOI:** 10.7759/cureus.112908

**Published:** 2026-07-18

**Authors:** Amena Fardous, Anusha Sivasuriam, Mohamed Khalifa

**Affiliations:** 1 Obstetrics and Gynaecology, Cwm Taf Morgannwg University Health Board, Merthyr Tydfil, GBR; 2 Obstetrics and Gynaecology, Prince Charles Hospital, Cwm Taf Morgannwg University Health Board, Merthyr Tydfil, GBR

**Keywords:** atypical presentation, conservative management, endometriosis and chronic pelvic pain, gynaecology emergency, site of infection

## Abstract

Endometriosis may rarely present with atypical manifestations that closely mimic intra-abdominal malignancy or severe pelvic sepsis, resulting in significant diagnostic uncertainty. We present two contrasting cases of presumed severe endometriosis with uncommon clinical and radiological presentations. In the first case, a woman in her 30s presented with acute abdominal pain, progressive abdominal distension, and respiratory compromise associated with massive haemorrhagic ascites and markedly elevated cancer antigen 125 levels. Cross-sectional imaging demonstrated extensive deep infiltrative endometriosis with haemoperitoneum and multiple extra-pelvic endometriotic deposits. In the second case, a woman in her 30s presented with a complex adnexal mass, para-aortic lymphadenopathy and inflammatory features initially concerning for tubo-ovarian abscess or underlying malignancy. Following prolonged antibiotic therapy, complete radiological resolution of the adnexal lesion was observed, with subsequent imaging findings considered highly suggestive of an infected endometrioma. These cases emphasise the diverse spectrum of endometriosis presentations, the limitations of non-histological diagnosis and the importance of multidisciplinary assessment, radiological expertise and longitudinal follow-up in complex presentations.

## Introduction

Endometriosis is characterised by the presence of endometrial glands and stroma outside the uterine cavity and affects approximately 10% of women of reproductive age [1]. The disease most commonly involves the ovaries, pelvic peritoneum, uterosacral ligaments and pouch of Douglas; however, atypical extra-pelvic and severe inflammatory presentations have been described [2]. Although pelvic pain, dysmenorrhoea and infertility are the most frequent manifestations, some patients present with uncommon complications, including haemorrhagic ascites, haemoperitoneum and infected endometriomas [3,4].

These atypical presentations may mimic advanced gynaecological malignancy or pelvic sepsis due to elevated tumour markers, complex adnexal masses and inflammatory changes on imaging [5]. Recent guidelines increasingly support imaging-based diagnosis of endometriosis in selected cases, particularly when characteristic magnetic resonance imaging findings are present [6]. However, histological confirmation remains the diagnostic gold standard.

We present two contrasting cases of presumed severe endometriosis presenting with unusual clinical manifestations and significant diagnostic uncertainty.

## Case presentation

Case 1

A woman in her 30s presented to the emergency department with sudden onset abdominal pain, progressive abdominal distension, nausea, vomiting and shortness of breath. Clinical examination demonstrated tense ascites with respiratory compromise. Computed tomography of the abdomen and pelvis demonstrated a bulky fibroid uterus with gross ascites and diaphragmatic splinting. Approximately 10 litres of ascitic fluid were drained.

Laboratory investigations demonstrated markedly elevated inflammatory markers and elevated cancer antigen 125 (CA-125), initially raising concern for an underlying malignancy. Ascitic fluid cytology did not demonstrate malignant cells; however, fluid culture grew Klebsiella species. Human immunodeficiency virus testing was negative, and acid-fast bacilli were not identified. She had no previous history of gynaecological malignancy or chronic liver disease.

Laboratory findings are summarised in Table 1.

**Table 1:** Laboratory investigations in Case 1

Investigation	Result	Reference Range
C-reactive protein	350 mg/L	<5 mg/L
CA-125	440 U/mL	<35 U/mL
Ascitic fluid cytology	No malignant cells	N/A
Ascitic fluid culture	Klebsiella positive	No growth

Magnetic resonance imaging of the pelvis demonstrated a large multifibroid uterus measuring approximately 14.4 × 10.8 cm with moderate ascites. Additional imaging demonstrated deep infiltrative endometriosis characterised by plaque-like fibrotic thickening at the torus uterinus, bilateral medialised “kissing ovaries”, haemorrhagic foci within the ovaries and multiple endometriotic deposits involving the umbilicus and sub-hepatic region.

**Figure 1:**
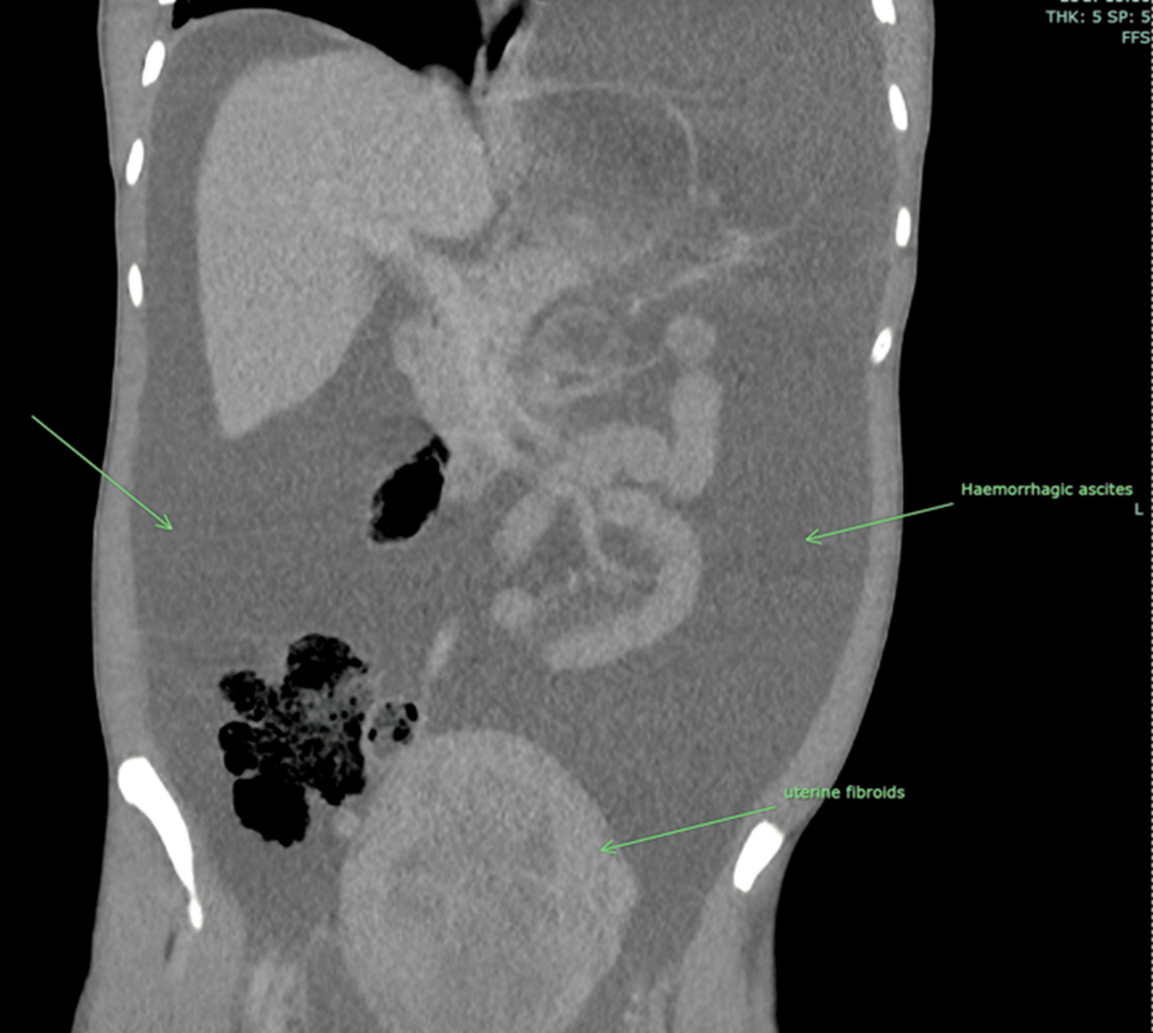
CT image demonstrating ascites and fibroid uterus in Case 1

The imaging findings were considered highly suggestive of severe deep infiltrative endometriosis with haemoperitoneum, as seen in Figure 1. The patient required intensive care admission due to right ventricular impairment associated with severe ascites and diaphragmatic compromise. Following multidisciplinary discussion, she was treated conservatively with intravenous piperacillin/tazobactam and hormonal suppression therapy using dienogest and Ryeqo (relugolix, estradiol and norethisterone acetate).

Computed tomography of the thorax additionally demonstrated right upper lobe atelectasis secondary to diaphragmatic splinting. The patient subsequently demonstrated significant clinical improvement with resolution of anaemia, reduction in pain and marked improvement in functional status.

**Figure 2:**
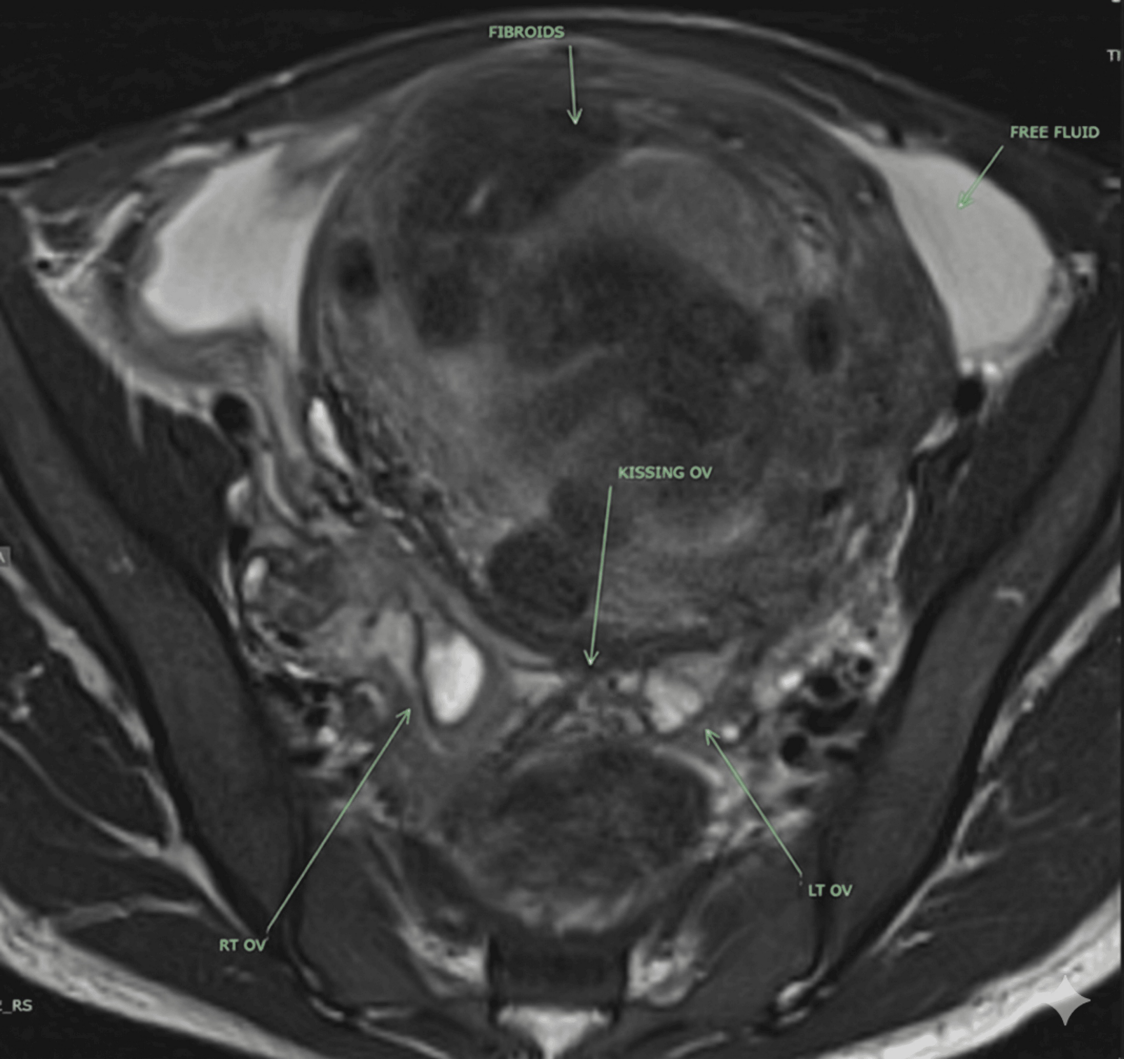
MRI showing kissing ovaries and posterior endometriotic plaque (annotated) in Case 1 after the treatment; ascites has resolved

Follow-up magnetic resonance imaging, shown in Figure 2, demonstrated interval reduction in the size of several uterine fibroids and complete resolution of pelvic free fluid. Persistent imaging findings of severe deep infiltrative endometriosis remained evident. Fertility counselling was undertaken due to concerns regarding future fertility and the potential risks associated with discontinuation of medical therapy. 

Case 2

**Figure 3:**
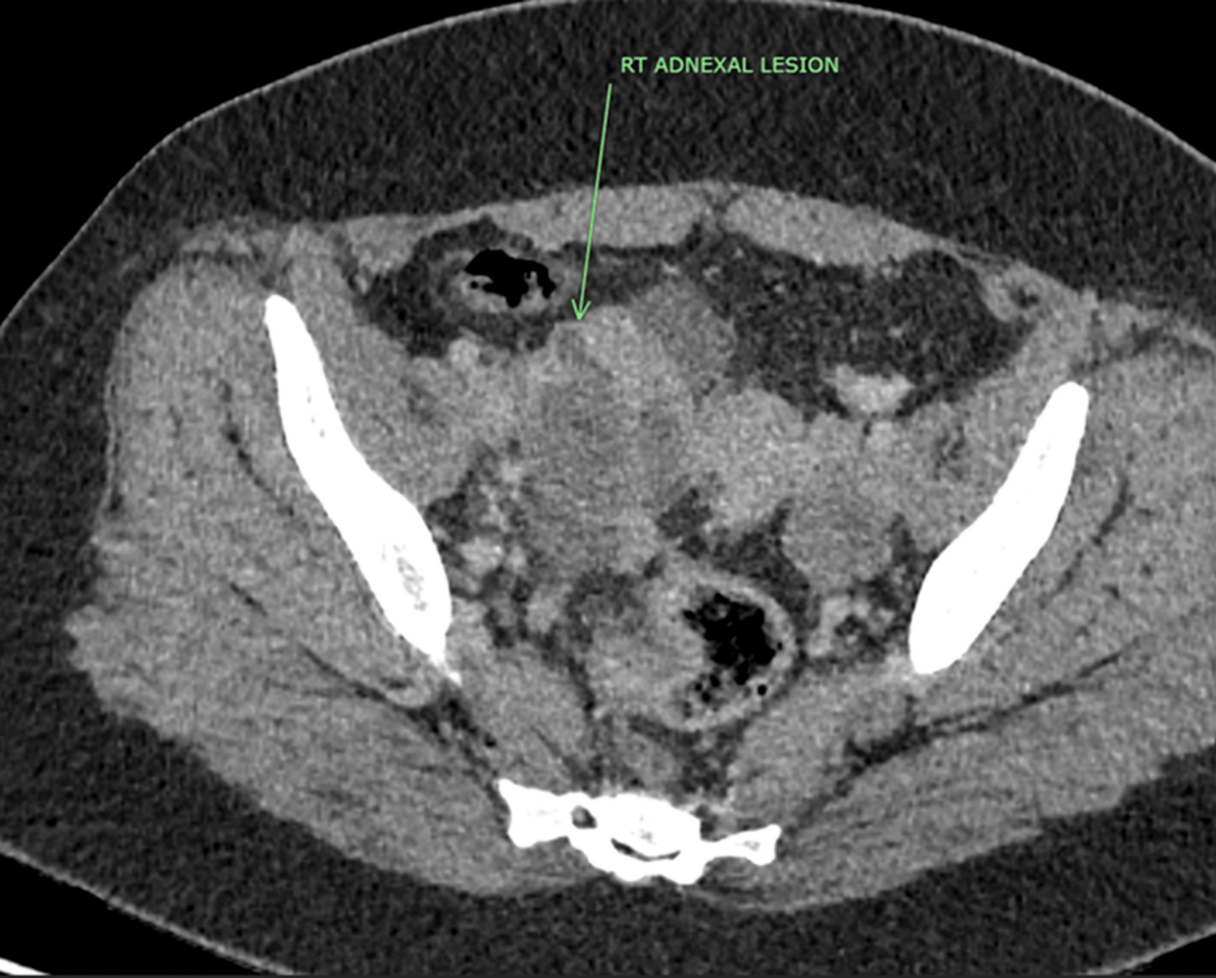
Right adnexal lesion suggestive of a tubo-ovarian mass in Case 2

A woman in her 30s was referred to gynaecology services following identification of a right-sided tubo-ovarian abscess associated with a complex adnexal mass. Initial imaging shown in Figure 3 raised concern regarding malignancy due to associated para-aortic lymphadenopathy and peritoneal nodules. The differential diagnosis included tubo-ovarian abscess, ovarian malignancy and infected endometrioma.

She was treated with prolonged courses of doxycycline and metronidazole and subsequently became asymptomatic. Repeat imaging demonstrated a reduction in the size of the adnexal lesion and interval improvement in para-aortic lymphadenopathy. 

Laboratory findings are summarised in Table 2.

**Table 2:** Laboratory investigations in Case 2

Investigation	Result	Reference Range
CA-125	100 → 23 U/mL	<35 U/mL
CA19-9	Normal	<37 U/mL
Carcinoembryonic antigen	Normal	<5 µg/L

**Figure 4:**
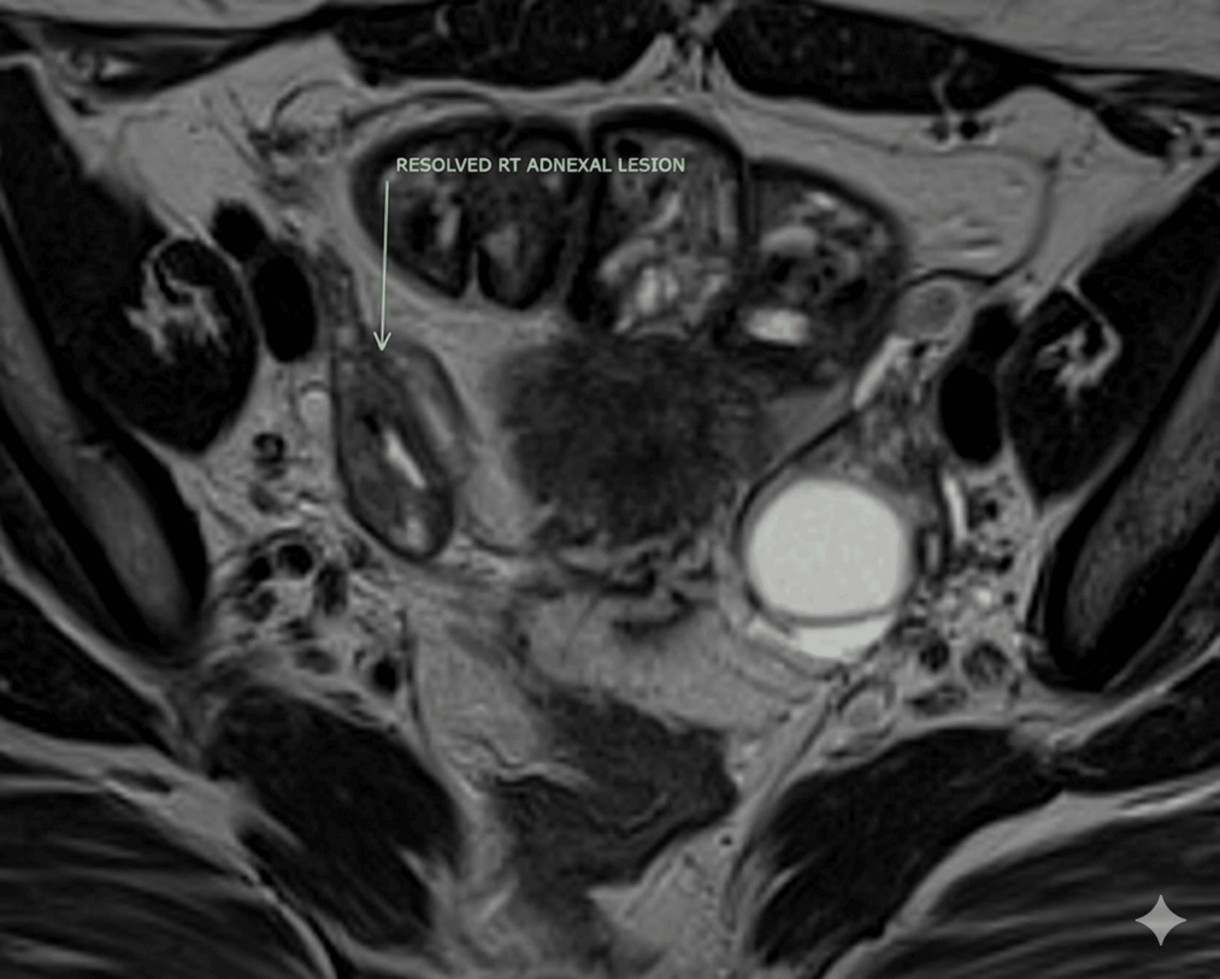
MRI of Case 2 showing the resolved adnexal mass and normal pelvic anatomy

Magnetic resonance imaging (Figure 4) subsequently demonstrated complete resolution of the previously identified adnexal mass. Residual findings included adenomyosis, a simple ovarian cyst and a lesion within the broad ligament, suggestive of endometriosis.

The patient had initially been scheduled for bilateral salpingo-oophorectomy due to concern regarding persistent pelvic sepsis and possible malignancy. However, following a multidisciplinary review of the interval imaging, surgery was cancelled because the adnexal lesion had completely resolved.

The overall radiological and clinical findings were considered highly suggestive of an infected endometrioma rather than malignancy. The patient was subsequently commenced on hormonal suppression therapy with dienogest. Due to medication intolerance, treatment was later transitioned to Ryeqo and eventually discontinued because of severe headaches.

Serial ultrasound surveillance demonstrated no recurrence of the adnexal lesion, and the patient remained clinically asymptomatic during follow-up.

## Discussion

These cases demonstrate the diagnostic complexity of atypical endometriosis presentations, particularly when associated with massive ascites, inflammatory changes and elevated tumour markers. Severe endometriosis may mimic advanced ovarian malignancy due to the presence of haemorrhagic ascites, complex adnexal masses and markedly elevated CA-125 levels [3,5].

Haemorrhagic ascites secondary to endometriosis is rare and remains poorly understood. Several mechanisms have been proposed, including repeated cyclical bleeding from peritoneal endometriotic implants, leading to chronic peritoneal irritation and inflammatory exudative fluid accumulation [7]. Deep infiltrative endometriosis may also result in extensive fibrosis and adhesions, contributing to complex radiological appearances.

In the first case, MRI demonstrated several classical features of severe deep infiltrative endometriosis, including kissing ovaries, haemorrhagic ovarian deposits and plaque-like fibrosis involving the torus uterinus. Although histological confirmation was unavailable, the radiological findings strongly supported the diagnosis according to contemporary imaging-based diagnostic criteria [6].

The second case posed a greater diagnostic challenge because imaging findings overlapped significantly with pelvic inflammatory disease and tubo-ovarian abscess. Complete radiological resolution following antibiotic therapy suggested a substantial infective component. Nevertheless, the presence of residual endometriotic features and the overall clinical context supported the possibility of an infected endometrioma.

Ultrasound remains the recommended first-line imaging modality for suspected endometriosis, particularly for ovarian endometriomas and deep infiltrative disease involving the pelvis [6,8]. Magnetic resonance imaging may provide additional anatomical detail in complex cases and is particularly useful when malignancy cannot be excluded.

Recent guidelines increasingly support non-invasive diagnosis of endometriosis based on characteristic imaging findings without mandatory surgical confirmation [6]. However, histopathological diagnosis remains the gold standard, and the absence of biopsy confirmation represents an important limitation in both these cases.

Endometriosis has additionally been associated with rare systemic and inflammatory manifestations. Autoimmune encephalopathy and ovarian tumours have been reported in association with severe endometriosis in rare circumstances [9]. Furthermore, untreated endometriomas may rarely predispose patients to intra-abdominal haemorrhage during pregnancy, highlighting the importance of close surveillance in women pursuing fertility treatment [10].

A limitation of these cases is the absence of histological confirmation. Although imaging findings strongly supported endometriosis in the first case, the second case remains diagnostically challenging because pelvic infection and endometriosis may demonstrate substantial overlap on imaging. Current international guidelines support an imaging-based diagnosis in appropriately selected patients with characteristic ultrasound or magnetic resonance imaging findings; however, the histopathological confirmation remains the diagnostic gold standard. The absence of tissue confirmation should therefore be recognised as a limitation when interpreting these cases.

## Conclusions

Endometriosis may present with rare and severe manifestations that closely resemble advanced malignancy or pelvic infection, posing substantial diagnostic and management challenges. These two cases illustrate the broad clinical spectrum of endometriosis, ranging from massive haemorrhagic ascites with haemoperitoneum to a presumed infected endometrioma presenting as a complex adnexal mass. In both cases, multidisciplinary evaluation and serial imaging played a central role in guiding management and avoiding unnecessary radical surgery. Although histological confirmation was unavailable, the clinical and radiological findings were highly suggestive of severe endometriotic disease. These cases highlight the importance of considering endometriosis within the differential diagnosis of atypical pelvic and intra-abdominal pathology in women of reproductive age and underscore the need for careful long-term follow-up, particularly in patients with fertility considerations. These cases also highlight that multidisciplinary review and serial imaging may prevent unnecessary surgery when imaging findings evolve over time, while recognising that histological confirmation remains the reference standard whenever feasible.
